# Olfactory Loss after Uvulopalatopharyngoplasty: A Report of Two Cases with Review of the Literature

**DOI:** 10.1155/2014/546317

**Published:** 2014-12-31

**Authors:** Rong-San Jiang, Yi-Hao Chang

**Affiliations:** ^1^Department of Otolaryngology, Taichung Veterans General Hospital, 1650 Taiwan Boulevard, Section 4, Taichung 40705, Taiwan; ^2^School of Medicine, Chung Shan Medical University, Taichung, Taiwan; ^3^Faculty of Medicine, National Yang-Ming Medical University, Taipei, Taiwan

## Abstract

Uvulopalatopharyngoplasty (UPPP) has been a popular surgical method for treating obstructive sleep apnea syndrome since it was introduced in the early 1980s. Olfactory loss has been reported as a rare side effect in several cases. However, the olfactory test results and the prognosis were not mentioned in these cases. We present two patients who complained of loss of olfactory function after UPPP. Their olfactory function was evaluated by the phenyl ethyl alcohol odor detection threshold test and the University of Pennsylvania Smell Identification Test. After treatment with steroid and zinc salt, their olfactory function was improved but not recovered completely.

## 1. Introduction

Recently, it has been shown that olfactory disorders occur at a higher rate than previously thought. The frequency of olfactory dysfunction was estimated to be 16% in one study [1]. The most common etiologies of olfactory dysfunction are sinonasal diseases, head trauma, and upper respiratory infection [2]. Other causes include congenital olfactory dysfunction, exposure to toxin, and surgical intervention [3]. Among surgical procedures, nasal surgery was the leading cause of olfactory dysfunction.

Uvulopalatopharyngoplasty (UPPP) was introduced as a surgical method for treating obstructive sleep apnea syndrome (OSAS) in the early 1980s [4]. Since then, it has become a popular surgical procedure for the treatment of OSAS and snoring [5]. The most common late side effects after UPPP are difficulty in swallowing, nasal regurgitation, pharyngeal dryness, and voice changes [6]. Other reported side effects included taste and smell disturbances and velopharyngeal insufficiency [7]. Olfactory loss after UPPP was reported in two studies, but neither study included olfactory test results [6, 8].

Herein, we report two patients who complained of olfactory loss after UPPP. Their olfactory function was measured by widely used olfactory tests and reevaluated after medical treatment.

## 2. First Case

A 36-year-old male came to our clinic on January 16, 2013. He complained of loss of smell shortly after undergoing UPPP for treatment of OSAS in June 2011. He thought his olfactory function was normal before UPPP. He had no history of head trauma or any upper respiratory infection before loss of smell but did have mild nasal obstruction. He had seen several physicians for help but no definitive treatment was given. He remained anosmic. The physical examination showed that the oropharynx was widely open after UPPP without any complication. The nasal endoscopy showed that the nasal structures were normal without any nasal secretion in the nasal cavity. The nasopharynx was without obstruction.

**Figure 1:**
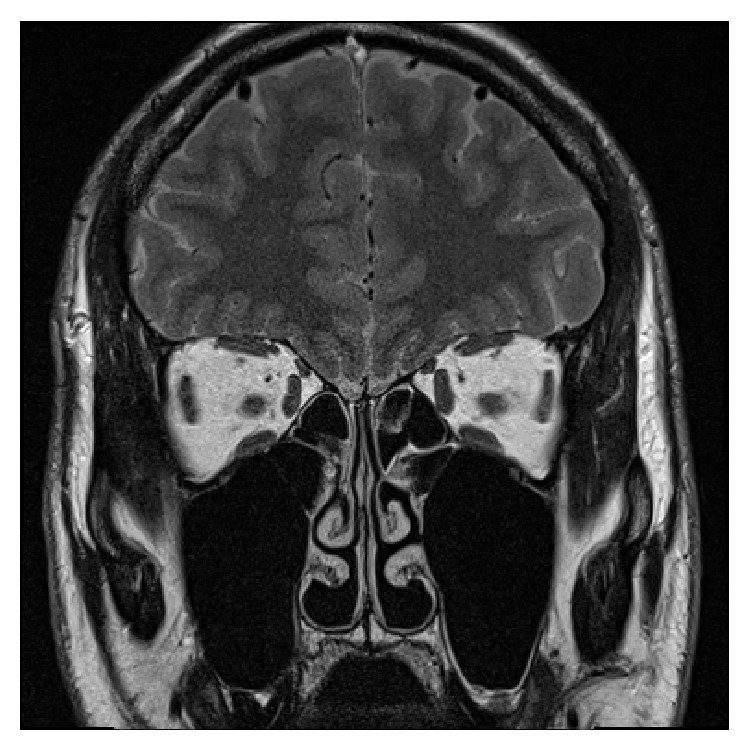
Magnetic resonance imaging showed normal sinus and brain structures.

On the first visit at our clinic, he received the phenyl ethyl alcohol (PEA) odor detection threshold test, and his olfactory threshold was −1. Magnetic resonance imaging showed normal sinus and brain structures (Figure 1). The volume of the right olfactory bulb was 24.25 mm^3^ and that of the left olfactory bulb was 25.57 mm^3^.

A course of high-dose prednisolone (1 mg/kg per day) with tapering for 2 weeks was given. Two months later, the patient found that he could detect some odorants but all of them smelled the same. The PEA odor detection threshold remained at −1. The score of the traditional Chinese version of University of Pennsylvania Smell Identification Test (UPSIT) was 16. One month later, the patient's PEA odor detection threshold improved to −3.25. The score of the traditional Chinese version of UPSIT was 19. Another course of high-dose prednisolone was given. Two months later, the patient said that his olfactory function improved a little more and his PEA odor detection threshold improved to −6.25, but the score of the traditional Chinese version of UPSIT was 15.

## 3. Second Case

A 33-year-old male came to our clinic on May 16, 2014. He complained of loss of smell shortly after undergoing UPPP for treatment of OSAS in October 2013. He thought that his olfactory function was normal before UPPP. He had no history of head trauma, upper respiratory infection before loss of smell, or any nasal symptom. The operator saw him several times but nothing was given to treat his smell problem. He remained anosmic. The physical examination showed the oropharynx was widely open after UPPP without any complication. The nasal endoscopy showed that the nasal structures were normal without any nasal secretion in the nasal cavity. The nasopharynx was without obstruction.

**Figure 2:**
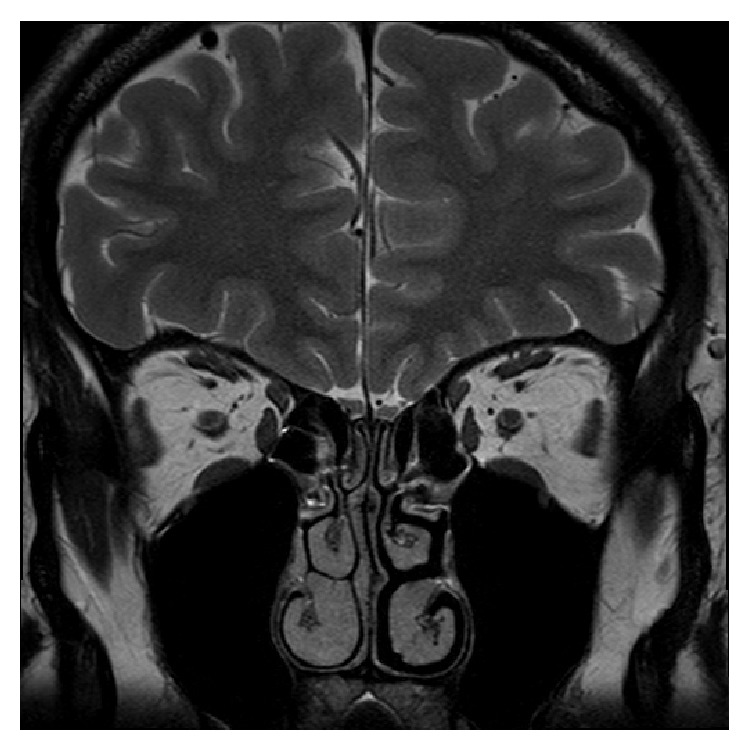
Magnetic resonance imaging showed mild sinusitis with normal brain structures.

He received the PEA odor detection threshold test, and his olfactory threshold was −1. Magnetic resonance imaging showed sinusitis with normal brain structures (Figure 2). The volume of the right olfactory bulb was 34.44 mm^3^ and that of the left olfactory bulb was 35.93 mm^3^.

A course of high-dose prednisolone (1 mg/kg per day) with tapering for 2 weeks was given. Two months later, the PEA odor detection threshold remained at −1. The patient was treated with zinc gluconate (10 mg t.i.d.) for a month, and he found that his olfactory function was improved. His PEA odor detection threshold was −3.875, and the score of the traditional Chinese version of UPSIT was 24. The patient was treated with zinc gluconate for two more months, and his PEA odor detection threshold improved to −6.25. The score of the traditional Chinese version of UPSIT was 25.

## 4. Discussion

Odor detection threshold test is one of the most commonly used olfactory tests [3]. Detection threshold tests are used to estimate the lowest concentration of a stimulus that can be detected. In the traditional PEA odor detection threshold test, a two-alternative forced-choice single-staircase detection threshold procedure is used [9]. It consists of the presentation of two glass sniff bottles to the subject. One contains 20 mL of a given concentration of PEA dissolved in light mineral oil, whereas the other contains the mineral oil alone. These two bottles are opened and positioned over the subject's nose in a random order. The subject indicates which bottle contains the stronger odor. If no difference is perceived, a guess is required. The test is completed when seven reversals are acquired. The geometric mean of the last four reversed points of the seven reversals is used as the threshold estimate. PEA concentrations range from 10^−1^ to 10^−9^ log vol/vol in half-log concentration steps. The normal detection olfactory function (normosmia) is defined as PEA detection threshold equal to or below −6, partial loss of detection olfactory function (hyposmia) is defined as PEA detection threshold higher than −6 but below −1, and total loss of detection olfactory function (anosmia) is defined as PEA detection threshold equal to −1.

The other most widely used of these tests is the UPSIT (2). It is an odor identification test. UPSIT has been administered to nearly one-half million patients [10]. Because of its wide applicability, the UPSIT has been translated into multiple language versions, including traditional Chinese version. The UPSIT and the traditional Chinese version of UPSIT (Sensonics, Inc., Haddon Heights, NJ) are comprised of four 10-odorant booklets that can be self-administered in 10 to 15 minutes. Each of the 40 “scratch and sniff” odorants is embedded in 10 to 50 *μ*m microcapsules fixed in a propriety binder and positioned on brown strips located at the bottom of the pages of each test booklet [11]. When the examinee takes the UPSIT or traditional Chinese version of UPSIT, he/she releases each of the 40 odorants by scratching the strip with a pencil tip in a standardized manner. The identity of the released odorant is signified by choosing a name from a set of 4 odor descriptors [10]. The test is scored as the number of odors identified correctly. A response is required for each test item even if no smell is perceived (i.e., the test is forced choice), allowing for the detection of malingering on the basis of improbable responses.

Olfactory dysfunction can be classified into two main types: conductive olfactory loss and sensorineural loss [1]. OSAS has been shown to have negative effects on olfactory function [12]. It has been suggested that altered nasal structure affects olfactory function in OSAS patients [13]. UPPP improved nasal breathing in many OSAS patients with impaired nasal breathing, so the olfactory function in OSAS patients might be improved by UPPP [13, 14].

The only large series that evaluated smell disturbances after UPPP was conducted by Hagert et al. [6]. Results of a questionnaire revealed that 22 of 292 patients felt that their olfactory function was impaired 2–8 years after UPPP. They considered that olfactory loss after UPPP seemed hard to explain by air flow changes only. Nasopharyngeal stenosis is a rare complication of UPPP [15]. It might change air flow through choanae and impair olfactory function, but nasopharyngeal stenosis was not observed in both of our cases by nasal endoscopy.

Our patients presented as anosmic 6 months after UPPP. After prednisolone and zinc treatment, their olfactory function improved progressively, as shown by the results of the PEA detection threshold test. However, the UPSIT showed that their identification ability remained unchanged. If olfactory loss is conductive type, systemic steroid therapy usually has a good effect on both detection and identification ability [16, 17]. In conclusion, the risk of olfactory loss should be discussed with patients who are going to undergo UPPP.
